# Strategies for Psychiatric Rehabilitation and their Cognitive Outcomes in Schizophrenia: Review of Last Five-year Studies

**DOI:** 10.2174/1745017902117010031

**Published:** 2021-05-24

**Authors:** Antonio Rampino, Rosa M. Falcone, Arianna Giannuzzi, Rita Masellis, Linda A. Antonucci, Silvia Torretta

**Affiliations:** 1 Department of Basic Medical Sciences, Neuroscience, and Sense Organs, University of Bari Aldo Moro, 70124 Bari, Italy; 2 Azienda Ospedaliero-Universitaria Policlinico di Bari, 70124 Bari, Italy; 3 Department of Education, Psychology and Communication, University of Bari Aldo Moro, 70122 Bari, Italy

**Keywords:** Schizophrenia, Cognition, Psychiatric Rehabilitation, Cognitive Remediation, Outcomes, Individualized intervention

## Abstract

**Background:**

Cognitive deficits are core features of Schizophrenia, showing poor response to antipsychotic treatment, therefore non-pharmacological rehabilitative approaches to such a symptom domain need to be identified. However, since not all patients with Schizophrenia exhibit the same cognitive impairment profile, individualized rehabilitative approaches should be set up.

**Objectives:**

We explored the last five-year literature addressing the issue of cognitive dysfunction response to rehabilitative methodologies in Schizophrenia to identify possible predictors of response and individualized strategies to treat such a dysfunction.

**Conclusion:**

A total of 76 studies were reviewed. Possible predictors of cognitive rehabilitation outcome were identified among patient-specific and approach-specific variables and a general overview of rehabilitative strategies used in the last five years has been depicted. Studies suggest the existence of multifaced and multi-domain variables that could significantly predict pro-cognitive effects of cognitive rehabilitation, which could also be useful for identifying individual-specific rehabilitation trajectories over time.

An individualized rehabilitative approach to cognitive impairment in Schizophrenia is possible if taking into account both patient and approach specific predictors of outcomes.

## 1 INTRODUCTION

Between the late 1960s and early 1980s, the de-institutionalization process brought patients affected by a psychiatric disorder to move from hospitals to the real world, thus raising the need for a Psychiatric Rehabilitation (PR) approach to re-integrate them into their families, workplaces, and society in general. Since then, our understanding of psychiatric disorders has importantly improved, and PR has enriched its original social function with the development of diagnosis- and patient-specific strategies. With this regard, in the last few years, the tremendous development of individualized interventions, including those in PR, has been registered in the treatment of severe psychotic disorders, including Schizophrenia (SCZ).

Indeed, SCZ is one of the most debilitating psychiatric conditions because of its disabling psychopathology, which includes Positive Symptoms (delusions and hallucinations and Negative Symptoms (social withdrawal, apathy, alogia, anergia, and similar) and because psychopathology is often complicated by cognitive decline. In fact, SCZ cognitive performance is overall1.5-2 standard deviations poorer than those exhibited by Healthy Controls (HC) at the overall cognitive level [1]. Furthermore, cognitive deficits in SCZ appear in a wide variety of neuropsychological domains, spanning from Working Memory (WM) to selective attention, speed of processing, and social cognition [2]. Notably, cognitive impairments affect patients’ ability to process even very simple stimuli, thus dramatically lowering their Quality of Life (QoL) and global functioning. Finally, none of the SCZ-related cognitive impairments has proven responsiveness to Antipsychotic (AP) drugs [3, 4], currently the only pharmacological remedy approved for the treatment of the disorder. This has made non-pharmacological PR approaches extremely attractive for both research and clinical practice.

Therapeutic strategies specifically targeting cognitive dysfunction of SCZ are collectively indicated as Cognitive Remediation (CR) and involve methodologies pointing at improving cognition by directly or indirectly enhancing brain cognitive capacity. However, not all SCZ patients exhibit the same cognitive impairment profile, and especially not in the very same domains [5], suggesting that an individualized approach to CR intervention in SCZ is highly desirable –probably even necessary- and that a switch from a group-level perspective to an individualized, single-subject level perspective [6, 7] is required. Importantly, while a number of studies have focused on the effectiveness of CR approaches to SCZ cognitive impairment, very few have reported on the cognitive outcomes of PR strategies not directly targeting cognition. Indeed, such strategies may impact cognitive improvement by affecting other variables, e.g.,psychological-physical health, social life, and QoL as a whole. Therefore, current evidence suggests that a Precision Medicine approach to the management of cognitive impairment in SCZ, while being tailored to each patient’s cognitive profile, should target multiple factors instead of a single one.

Here, we conducted a systematic review of the last five-year scientific literature investigating cognitive outcomes of rehabilitative strategies that directly (CR approaches) and/or indirectly (overall PR approaches) impact cognition in patients with SCZ. We aimed at identifying clinical and non-clinical variables predicting the cognitive outcome of such strategies in order to ultimately provide all professionals working with SCZ patients with a number of tools of potential usefulness in the management of cognitive symptoms of this disabling disorder.

## 2 METHODS

### 2.1 Search Strategy (Electronic Databases, Search Terms, Hand-searches)

Literature collection was performed following the guidelines provided by the PRISMA protocol [8]. A literature search was conducted using the PubMed search engine (https://www.ncbi.nlm.nih.gov/pubmed/), which primarily accesses the MEDLINE database of scientific reports, and the American Psychological Association database APA PsycNet (https://psycnet.apa.org/search). Different study types were included: 1) feasibility studies; 2) case reports; 3) qualitative studies; c) surveys; d) cohort studies and e) randomised controlled trials. Previous reviews and meta-analyses on this topic were excluded. The following search terms were used: “Schizophrenia” AND “Rehabilitation” AND “Methodologies” AND “Cognition”.

The current review was registered within the International Prospective Register of Systematic Reviews (PROSPERO, Registration Number: CRD42020201569).

### 2.2 Eligibility Criteria

Inclusion criteria were: a) publication date between 01/01/2015 and 01/08/2020; b) articles including individuals diagnosed with SCZ-spectrum disorders; c) English language; d) reference to any rehabilitative intervention whose primary or secondary outcome was cognition or cognitive functioning.

Exclusion criteria were: a) abstract-only publication; b) articles not including the investigation of any cognitive outcome; c) languages different from English.

### 2.3 Study Selection, Data Extraction, and Synthesis

The authors operated a blind research process. A publication was included when the independent reviewers agreed that all eligibility criteria were satisfied. All relevant information related to the topic of the current review was extracted and collected in a pre-determined template form, whose structure was shared by all authors beforehand. The literature quality check was performed following procedures suggested by Pawson *et al.* 2005 (9).

A flowchart of the review process is reported in Fig. (**1**).

## 3 RESULTS

### 3.1 Predictors of CR and PR Cognitive Outcomes

Table **1** reports a general overview of the studies included in the current review and their main outcomes. A total of 76 articles were included in the review process. The average sample size was N=72.65 ±12. Notably, such esteem is potentially inflated by the heterogeneity of diagnoses which in most cases included, along with Schizophrenia-spectrum disorders, broader diagnosis of “psychosis”, Not Otherwise Specified Psychoses and, in some cases, Major Depressive Disorder, Bipolar Disorder, and further psychiatric diagnoses, such as Anxiety Disorders, Obsessive-Compulsive Disorder, and Personality Disorders. Additionally, it is possible that sample size esteems were affected by unexplored-implicit variables, such as poor active participation in protocols and unassessed dropouts. Indeed, studies report that participation of patients with SCZ to study protocols is poor as a consequence of their clinical state, cognitive impairment, and medication side effects [10], thus representing an important limit to appropriate assessment of outcomes. Consistently, the studies we examined reported that active participation in rehabilitation procedures was particularly affected by both Negative Symptoms (including apathy, anergia, abulia, social withdrawal, and lack of interest for most aspects of life) and other clinical variables, such as the severity of positive and depressive symptoms, as well as the overall cognitive ability of individuals.

Among the included studies, few ones provided either qualitative or quantitative esteem of patients’ participation as predictors of cognitive outcomes, and only some of them targeted “motivation to participate” as a target of the rehabilitative intervention itself. Specifically, Bryce and collaborators (Bryce *et al.*, 2018] [11] ran a Single-blind Randomized Controlled Trial (RCT) aiming at investigating whether baseline (‘early’) task-specific Intrinsic Motivation (IM), assessed through the Intrinsic Motivation Inventory for Schizophrenia Research (IMI-SR) [12], predicted session attendance to a CR protocol administered through the software CogPack [13]. Furthermore, the authors investigated whether baseline or changes in task-specific IM levels predicted cognitive improvement. They found that patients’ early perception of task value/usefulness explained a significantly higher portion of the variance in total session attendance compared with the variance explained by baseline cognitive and clinical variables. Furthermore, it remained the only significant multivariate predictor of total session attendance over time. Moreover, increases in the levels of interest/enjoyment and recognition of the value/usefulness of the CR protocol were significantly associated with post-CR cognitive improvement. Overall, in the vast majority of CR protocol completers, the authors found an increase in IM, which could suggest that on the one hand, IM could predict CR-related cognitive outcomes, and on the other hand, IM is susceptible to CR-related therapeutic effects.

Rather consistently, in a retrospective study investigating the effectiveness of a Computer-Assisted CR (CACR) approach on cognition, QoL, and self-esteem within a 12-month follow-up period, Garrido and collaborators [14] reported that patients undergoing CACR improved significantly more than the control group in processing speed, working memory, reasoning, and problem-solving abilities, as well as QoL and self-esteem levels. Furthermore, compared to patients in the control group, those undergoing CACR showed greater improvement on the intrapsychic foundation sub-scale of the Quality of Life Scale QLS [15], a measure of intrinsic motivation in taking part in the rehabilitative program, thus providing further support to the evidence that CR intervention may enhance motivation.

Many studies have also highlighted how cognitive outcomes of CR and PR in SCZ may be conditioned by the mutual relationship between cognitive dysfunction and the clinical severity of the disorder. As previously reported, SCZ symptom severity may impact patients’ motivation to participate in rehabilitative procedures, thus indirectly limiting the chance of success of these procedures. Moreover, previous studies [16] have shown that clinical and cognitive manifestations of SCZ share, at least in part, a common neurobiological background, so that the degree of cognitive impairment and the severity of symptoms can be strictly related. Quite consequently, the majority of CR and PR studies we examined provided both complete cognitive and clinical pre- and post-treatment assessments.

According to the studies included in this review, tools adopted for cognitive assessment are quite standardized across studies and involved tests and scales included in the MATRICS Consensus Cognitive Battery (MCCB) [17], as well as in the Wechsler Adult Intelligence Scale – III(WAIS-III). Other domain-related fairly employed within the included studies were the Rey Auditory Verbal Learning Test (RAVLT), the Wisconsin Card Sorting Test (WCST), the Trail Making Test version A and B (TMTA and TMTB), the Continuous Performance Test – (CPT), and the N-Back Working Memory. On the other hand, pre- and post-treatment clinical assessments have been carried out mainly with the Positive and Negative Syndrome Scale (PANSS) [18], the Scale for the Assessment of Negative Symptoms (SANS), the Scale for the Assessment of Positive Symptoms (SAPS) [19], the Psychosis Evaluation tool for Common use by Caregivers (PECC) [20], along with further instruments used to assess more specific clinical domains, such as the Intrinsic Motivation Inventory for Schizophrenia Research (IMI-SR) [12] for the assessment of motivation and the Psychiatric Emergency Services (PES) [14], and the Acute Psychiatric Unit (APU) [14] for the assessment of hospital admission and re-admission.

Notably, studies showed that cognitive rehabilitation treatments may positively impact symptoms of SCZ. In particular, NegativeSymptom severity seems to be modulated by changes in cognitive functioning [21-26]. Consistently, a recent network meta-analysis [27] demonstrated a small-to-moderate effect of CR on Negative Symptoms (effect size g = − 0.30], although Negative Symptoms have not been considered a primary target for cognitive remediation. This could potentially suggest that the poor responsiveness that Negative Symptoms show towards currently used AP treatments may be, at least in part, overcome using non-pharmacological rehabilitative approaches. The opposite seems true for Positive Symptoms, which show relatively higher responsiveness to AP medication [28] but poorer if any, susceptibility to CR. Such evidence is in line with reports that common patterns of brain activity subtend cognitive impairment and negative, but not positive, psychopathology. With this regard, a recent review of 58 studies about the associations between neurocognitive impairments and psychotic psychopathology has documented that negative and disorganized dimensions of SCZ are associated with cognitive deficits, whereas positive and depressive dimensions are not and that association patterns correspond to shared underlying neurobiological pathways [16]. Not in line with this evidence, few studies reported poor or no effect of cognitive rehabilitation on both positive and negative symptom severity/course [29-34]. Among variables potentially impacting on cognitive outcome of CR and PR, importance has been given to age and duration of illness (DOI) at the moment of the beginning of the rehabilitation program. The age of participants to studies we examined ranged between 18 and 65, with few works including patients with a maximum age of 35, 40, 50, and 60 and one single study including the minimum age of 20.

Not all studies included DOI at the moment of the beginning of the study as a variable of interest. Where assessed, such a variable ranged from a minimum of 1 year to a maximum of 30 years. However, most studies considered 5-year illness length as a cut-off value to discriminate “chronic” from“early course” patients. One study [33] specifically focused on the effect of age, DOI, and their interaction on response to a computer-based Cognitive Remediation (CR) program compared with a control treatment. The authors found that patients in the CR group had greater improvements in attention and verbal WM, relative to those in the control condition. Furthermore, superior benefits of CR on cognitive outcomes were registered in younger patients with shorter DOI, suggesting that a different type of CR may be needed for older patients in the late stages of the illness for improving cognitive outcomes. Other studies reported an independent main effect of age or DOI on cognitive outcomes of rehabilitation. For example, Spangaro and collaborators [35] reported an effect of age on “cognitive flexibility”, a measure of response to a program including a CR Therapy (CRT) as an add-on to a Standard Rehabilitation Therapy (SRT), compared with an SRT-stand-alone paradigm, with younger age predicting better performance. Additionally, Rakitzi and collaborators [30] reported that, when comparing the response to an Integrated Psychological Therapy (IPT) and response to a Treatment As Usual (TAU) approach, “clinical insight”, another indicator of cognitive response, was higher in IPT patients as compared to TAU ones. Furthermore, among patients belonging to the IPT group, higher clinical insight was registered in those with shorter DOI.

Of note, many studies did not report any specific effect of either age or DOI on cognition, while some others did not include such variables as covariates of no interest in statistical models at all.

Based on the general principle that the effects of rehabilitation on cognition might lay on neuro-plasticity [36, 37], many studies have also focused on the importance of rehabilitative session frequency and duration as a factor directly or indirectly influencing neuronal structural and/or functional rearrangements over time. The number of rehabilitation sessions per time unit and duration of each session were quite different across studies we examined, depending on the specific type of rehabilitative approach and study design. However, most studies used the scheme of 1to3 sessions per week, with each session lasting 50 to 60 minutes and an overall average intervention duration of 12,6 weeks. The maximum duration was 26 weeks, the maximum number of sessions was 20 in two weeks and the maximum session length was 2 hours.

Fardig and collaborators [38] reported that increasing session frequency of an Illness Management and Recovery Program (IMR) progressively reduces the detrimental impact of neurocognitive dysfunction on illness self-management skill acquisition. Similarly, but within a different rehabilitative paradigm, Kumar and collaborators [39] showed that intensity (number of sessions per time unit) of a CR program was correlated with the program effectiveness both in a home-based and a clinic-based context, even in chronically ill patients. The authors concluded that the maximum frequency of sessions per week, which was higher than the usual one (6 sessions, instead of 2–3 sessions a week), might have had a significant impact on the process of neural plasticity supporting cognition.

Thus, on the one hand, included studies would suggest that some clinical and experimental variables may be used as predictors of response to CR and PR, thus paving the path to a possible individual-specific rehabilitative approach to cognitive dysfunction in SCZ. On the other hand, it is still unclear how durable over time cognitive effects of CR and PR are. With this regard, a meta-analysis of 39 studies on more than two thousand patients with SCZ published in 2011) concluded that combining CR and PR may expand CR benefits on cognition to patient’s global functioning and prolong them to significant durability over time. Among the studies we examined, two works specifically focused on the issue of durability of cognitive and non-cognitive outcomes of rehabilitative strategies. The first one [41] explored the persistence of both cognitive and functional effects of a combined Cognitive Remediation Therapy (CRT) and Standard Rehabilitation Therapy (SRT) 5 years after having completed the intervention, and found that the improvements in cognition persist at the medium-long term, while daily functioning improvements may require a longer training in order to be durable over time. The same group [42] explored the effect on cognition of a 3-month Computer-Assisted Cognitive Remediation (CACR) approach followed by a 3-month STR compared to a 6-months stand-alone CACR. Researchers found a significant global change in cognition at three months for patients in both groups. However, when investigating the presence of further improvements from three to six months program duration, a significant change only for executive functions in the group of patients treated with 6-months CACRwas observed. All other cognitive functions assessed, including working memory, psychomotor activity, verbal memory, and fluency, did not show any additional benefit from CACR longer duration. The authors concluded that post-training cognitive improvements may reach a plateau within 3 months of treatment, and then remain stable over time, with no significant progressions. Quite intriguingly, among the executive function domain, planning was the one showing greater improvement after CACR, suggesting that higher cognitive functions, such as planning itself, that are supported by a broad neural network also involving other specific and lower-level cognitive functions (*i.e.*, memory and attention), may benefit from longer cognitive rehabilitation treatments. Another study [43] compared the durability of CR and TAU pro-cognitive effect after 1 year from treatment completion and found that, while in the short term, global cognitive improvement reached with CR is greater than the one obtained with TAU; in the long-term, the only improvement in processing speed is maintained after 1 year. Quite surprisingly, while Working Memory (WM) was the cognitive function on which short-term effects of CR were more remarkable, in the long-term period, the effectiveness of CR and TAU on WM became overlapping, showing that timing of interventions is critical to rehabilitation of specific cognitive domains. Furthermore, the authors found that, differently from cognitive functioning, overall global functioning improvement was not different between the CR and the TAU groups in the short term, but differences in favor of CR emerged in the long-term observation, suggesting that functional effects of CR maybe not always detectable after the completion of treatment, but may evolve over time.

### 3.2 Main Rehabilitative Approaches

While looking at potential predictors of response, the effort to set up individualized strategies for the rehabilitative management of cognitive impairment in SCZ should take into account the large diversity of both CR and PR intervention that emerged in the last few years.

#### 3.2.1 Cognitive Remediation

Among approaches reported in the included studies, CR is the most broadly adopted. Indeed, such an approach is considered a stand-alone toolbox, since it includes all strategies and tools that have specifically been set up in order to target cognitive functioning.

CR strategies use both computer-based (Computer-Assisted Cognitive Remediation or CACR) or paper and pencil programs in order to stimulate cognitive abilities [44]. Some programs also teach the patient strategies for coping with the impact of cognitive impairments on psychosocial functioning [45]. A number of tools, including CogPack, REPYFLEC, Michael’s Game, JScores Training, CogRehab, CogREM, REHACOP, CogReHab, NEAR, Brain HQ, have been used in CR approaches. In the majority of studies, CR treatments have proven higher effectiveness than TAU approaches or other PR strategies, as previously highlighted.

#### 3.2.2 Perception-based Strategies

Evidence report that patients with SCZ suffer from visual perception alterations, such as low visual acuity [46], contrast sensitivity [47], and mid-level visual processes, along with perceptual organization deficits, including figure-ground segmentation, coherent motion detection, contour integration, and shape completion alterations [48]. Furthermore, specific visual-processing alterations in patients with SCZ are significantly related to poorer performance at cognitive tasks [49] and to impaired social cognition, including facial and emotion recognition [50]. This evidence could potentially suggest that therapeutic strategies that directly target the visual-processing impairments could improve cognitive and social functioning, in addition to improving visual functions [51].

Few studies have evaluated the effects of perception-based strategies in SCZ, and only some research groups have now explored the efficacy of computer-based cognitive remediation that includes visual training modules. In particular, Fisher *et al.* [52] reported that individuals with SCZ who completed training with both visual- and cognitive-control modules improved to a significantly greater extent cognitive abilities in processing speed, verbal learning, memory, and cognitive control domains, as well as in global cognition, compared with patients undergoing control procedures. Another RCT combined auditory, visual, and emotion-identification modules, and showed significant treatment-related improvements in source memory and verbal working memory in those who underwent this type of training [53].

#### 3.2.3 Psychosocial Training

Psychosocial treatments (skills training, family interventions, supported employment, assertive community treatment) share the common conceptual basis of “personal recovery”, *i.e.*, the attainment of a fulfilling and valued life [54]. Various studies have shown that psychosocial treatments have positive effects on cognition along with disease symptoms, treatment compliance, rehospitalization rates, quality of life, social cognition, and social functioning [55-58].

Among Psychosocial interventions, Assertive Community Treatment (ACT) is a rehabilitative approach based on the principle that a patient’s resistance to participate in rehabilitative programs can be overcome by moving the intervention setting to his/her home, and by engaging larger work staff and community services [59]. Studies reported that ACT may be useful to stabilize patient housing in the community, reducing hospitalizations, thus improving cost-benefit rate [60], while reducing symptoms [61]. Therefore, even though none of the studies we examined specifically addressed the issue of the effectiveness of ACT on cognition, benefits in cognitive function might be indirectly related to the proven impact of this approach on psychopathology.

Another approach used in Psychiatric Rehabilitation is Family Psychoeducation [FPE], a set of methodologies aimed at developing a collaborative relationship between the patient, his/her family, and the treatment team. Although these models differ in their theoretical background (cognitive behavioural versus broad-based systems theory) and modality (single-family versus multiple-family versus combined), they all share a series of characteristics, such as their delivery by mental health professionals, who teach strategies to reduce stress which ultimately aim at enhancing communication and problem solving [62]. Even though no specific effect of Family Psychoeducation on cognition has been reported by our included studies, an indirect effect of such strategy on cognitive functioning could be hypothesized as a consequence of its positive impact on the reduction of SCZ relapses and hospitalizations [63].

Psychosocial Training also includes interventions aimed at improving employment possibilities and work conditions in patients with SCZ. Indeed, work satisfaction is related to a range of benefits, including improvements in self-esteem, a sense of purpose, and reductions in symptoms [64]. Supported Employment (SE) and Vocational Rehabilitation (VR) are the main rehabilitative approaches used in this field. The most well-standardized and empirically validated model of SE is the Individual Placement and Support (IPS) model [65]. IPS focuses on the rapid search for competitive jobs integrated with specific support to facilitate optimal work performance [64]. A meta-analysis of 15 RCTs comparing competitive work achievement in IPS-SE as compared to traditional VR approaches indicated the superiority of IPS-SE with an effect size of 0.77 [66]. Few studies have also focused on the impact of IPS and VR on changes in cognitive performance and, while some works have highlighted that cognitive functioning may impact employment success rates and work performance [67]; [68]; [69]; [70], positive effects of VR and IPS strategies in addition to CR or as a stand-alone intervention on different areas of cognitive functioning have been reported [71].

Within a psychosocial approach to cognitive dysfunction in SCZ, the Social Skills Model [72] posits that social cognition, along with social perception, is critical to social functioning. Therefore, such a model adopts a number of rehabilitative strategies especially targeting social cognition, the most adopted of which is the Social Skills Training (SST] approach. With this regard, while two meta-analyses [73], [74] provided evidence that SST is effective on skills acquisition, social functioning, daily living, and psychopathology, still there is a lack of evidence that cognitive functioning may directly benefit from SST. Nonetheless, previous studies [75] reported that cognitive functioning may indirectly benefit from social functioning improvement, thus encouraging a multidisciplinary approach to cognitive rehabilitation in which pure cognitive remediation strategies are combined with an SST.

#### 3.2.4 Music/Movie/Art Intervention

The British Association for Art Therapy (BAAT) defined art therapy as “a form of psychotherapy that uses art media as its primary mode of expression and communication” to support people in distress [76]. People with SCZ often experience social withdrawal and difficulty in relating to each other. Art offers a safe and indirect means to connect with oneself and others and help people express and project their emotional and cognitive experiences [76]. Rather consistently, studies [77, 78] have shown that art therapies can promote recovery in SCZ.

Music intervention is known to significantly improve SCZ symptoms [79-82]. Additionally, a recent work using functional Magnetic Resonance (fMRI) to assess the impact of music intervention on neural circuits in patients with SCZ found that such an intervention can modulate activity and connectivity of brain circuitries subtending emotion and cognitive functioning, thus resulting in emotional behaviour and cognition improvements in patients with SCZ [80].

In recent years, the use of movies from a CR-PR perspective has also been taken into account [24]. In fact, cinema is an art form that integrates, exploits, and promotes cognitive functions, since the ability to follow and understand a movie requires WM, visuospatial capacity, phonological and multisensory competency, episodic memory, mental management of time, attention, management of temporo-spatial coordinates, and social empathy with the characters [83].

In literature, few works have studied art intervention approaches based on movie watching within PR strategies targeting SCZ. Gelkopf *et al.* reported benefits in SCZ psychopathology, patient state of mind, and social abilities after cinema therapy [84]. Furthermore, other groups [24] have developed structured methods based on cinema and TV series analysis [85] to induce CR benefits [86-88].

#### 3.2.5 Yoga

Yoga is a traditional Indian practice comprising alternative and complementary medicine approaches that have been evolving over the past 5000 years. The main components of yoga are: Asanas (physical postures), Pranayama (regulated breathing], and Meditation. Yoga Asanas are largely isometric positions that have been demonstrated to stimulate red-slow twitch fibres (type-I) [89] favouring physical and psychological states useful to meditation.

With regard to psychiatric rehabilitation, yoga has been adopted in the non-pharmacological treatment of many major psychiatric disorders, such as major depression [90], anxiety [91], obsessive-compulsive disorder [92], along with SCZ [93]. The combination of yoga therapy and conventional psychiatric medications showed numerous benefits across several clinical and cognitive SCZ core domains, including negative symptoms, socio-occupational functioning, and facial emotion recognition [94]. To our knowledge, so far, only one RCT showed improvement in accuracy indices of face memory, abstraction, mental flexibility, and speed index of attention, after combined yoga and physical exercise interventions, in association with drug therapy, in patients with SCZ [95]. Nonetheless, the pro-cognitive effects of yoga might be observed as an indirect consequence of its beneficial effects on SCZ negative symptoms.

#### 3.2.6 Physical Exercise

Regular physical activity has increasingly been used in the management of sedentary lifestyle, poor QoL, obesity, and a high risk for Metabolic Syndrome in patients with SCZ [96]. Additionally, physical exercise has proven effective in the management of SCZ symptoms [97-103] and quite recently has become part of non-pharmacological tools in pro-cognitive intervention in SCZ. So far, two studies have found significant increases in hippocampal volume in response to exercise [104, 105] in patients with the SCZ group, but not in HC. This suggests that exercise might attenuate the deterioration in hippocampal volume that occurs in the early phases of SCZ [104]. This is in line with similar results in aging populations, wherein hippocampus neuronal loss is attenuated by physical exercise [106].

Current recommendations for physical activity in SCZ suggest following the IOPTMH [International Organization of Physical Therapy in Mental Health] guidelines for aerobic and strength-based exercise to administer physical exercise-based training to SCZ patients [107]. Several authors have highlighted that training targeting SCZ should not be limited to a single type of physical exercise program [108, 109], thus recommending that different types of physical activities are included in training activities.

## 4 DISCUSSION

Cognitive impairment is a core aspect of SCZ which has got a detrimental impact on patients’ global functioning, social life, psychopathology, and overall QoL. In the last few years, studies have highlighted how such an impairment is supported by specific neurobiological alterations, at least in part also subtending specific clinical domains of the disorder [16, 110]. However, not all patients with SCZ manifest the same profile of cognitive impairment, suggesting that different patterns of brain dysfunction may support different profiles of cognitive alterations and possibly different degrees of susceptibility to treatments. With this regard, since the attempt to reduce SCZ-related cognitive dysfunction by use of AP medication has proven inconclusive [3, 4], efforts have been made to obtain patient-tailored rehabilitative strategies and identify possible predictors of such strategies’ pro-cognitive effects.

We have examined the last five-year literature addressing the issue of cognitive outcomes of CR and PR programs in SCZ. We found that a single-factor model in predicting such outcomes is not sufficient to capture the extreme complexity of cognitive recovery in SCZ and that the success of any rehabilitative approach to cognition depends on both individual-specific and approach-intrinsic variables. Among individual-specific predictors, importance has been given to patient’s clinical state at the moment of entering a rehabilitative program along with his/her degree of motivation to participate in the program itself. In particular, studies report that Negative Symptom severity has a critical impact on cognitive outcomes of CR and PR, possibly in virtue of a shared neurobiological background between cognitive function and negative symptomatology. This pivotal role of Negative Symptoms on intervention outcomes in SCZ is coherent with recent literature reporting that the burden of everyday difficulties reported by SCZ is positively associated with higher Negative Symptoms severity. Indeed, in SCZ, the presence of severe Negative Symptoms is associated with loss of functioning (even before the onset of disease) [111], with limited response to pharmacotherapies [112], and with lower perceived quality of life [113].

Possible speculation could be that Negative Symptoms may impact on cognitive outcomes of rehabilitative intervention by affecting the patient’s motivation to initiate and complete the corresponding program. Nonetheless, motivation is another critical variable to take into account and studies report that such variable may be affected by non-clinical patient-specific factors, such as personality aspects, including “intrapsychic foundation” [15], poor understanding of the value/usefulness of the rehabilitative program and poor insight [13]. Recent evidence [114] suggested that the relationship between Negative Symptom severity and motivation in SCZ may be partly explained by the social cognition deficits in SCZ, as these patients show decreased ability to anticipate pleasurable activities and to mentally visualize future events or intentions [115, 116]. This further testifies the importance of targeting socio-cognitive abilities into rehabilitation programs for SCZ.

Further individual-specific variables include patient age and duration of illness (DOI], with younger individuals having a history of shorter DOI responding better to rehabilitative programs than older patients with a chronic (generally longer than 5 years) DOI. This is consistent with findings from a previous contribution [117] highlighting that younger people undergoing CR had improvements in more cognitive domains than older people. Our findings strongly encourage CR/PR interventions to be as early and fast as possible, and further testify the need for adapting CR interventions to each recipient’s individualized characteristics, including age, to make them more effective. Indeed, a review of meta-analyses of studies exploring the topic of the effectiveness of cognitive remediation in SCZ [118] showed that early intervention in SCZ cognitive impairment may be considered as a tool to prevent or delay the onset of the illness in high-risk population and in subjects at the first episode of psychosis, while inducing important functional benefits in critical domains of the disorder, such as social functioning, employment, and role functioning.

Among individual-specific factors affecting response to cognitive rehabilitation, only one study [35] considered the role of genetics. In particular, the authors studied the interaction between type of rehabilitative treatment and genotype at the Excitatory Amino-Acid Transporter 2 (*EAAT2*) gene polymorphism rs4354668 (T/G) in a group of patients with SCZ undergoing either Cognitive Remediation Therapy (CRT) and Standard Rehabilitation Therapy (SRT) or SRT alone. They found that, regardless of treatment type, only individuals in the T/T genotype group had significant WM performance changes over the treatment time. Furthermore, such a group of individuals exhibited greater improvement in planning and cognitive flexibility when belonging to the CRT- and -SRT arm of the study than when belonging to the SRT – standalone one.

Among approach-intrinsic variables, importance has been given to intervention timing, including the overall intervention duration, the session frequency, and length. Most studies adopted a paradigm foreseeing a 12-week duration with 2to3 sessions per week, each one lasting 30 to 60 minutes, but variability in timing options is quite high due to diversity in intervention procedures and goals [40]. Furthermore, studies have also suggested combining CR with PR in order to prolong the pro-cognitive effects of CR while expanding them to the patient’s global functioning and daily life achievements [118].

Both individual-specific and approach-intrinsic factors may lay on the same neurobiological mechanism, that is, neuroplasticity. Indeed, such a mechanism could be a mediator of both age and DOI impact on CR and PR outcomes because of the known effects of aging and time on brain plasticity supporting cognition [119]. Similarly, neuroplasticity may mediate the reported effects of *EAAT2* genetic variation on cognitive outcomes of CR. In fact, while previous studies have highlighted the impact of genetic factors on neuroplasticity processes subtending cognition in SCZ [120], the beneficial effect of *EAAT2* rs4354668 T/T genotype on cognitive outcomes of CRT may be related to the decrease of *EATT2* brain expression in patients with this genotype, which reduces the potential excitotoxic damage on brain plasticity mediated by this gene [35].

On the other hand, neuroplasticity may mediate the effects of intervention timing and intensity on the quality of CR and PR outcomes. In fact, studies [121] have underscored training-related brain neuroplasticity changes highlighting the importance of a systematic stimulation of the brain in order to rehabilitate cognitive skills and some authors have emphasized the importance of intervention intensity in increasing pro-cognitive effects of rehabilitation. Not surprisingly, quite recently, “neuroplasticity-based” interventions have been developed as an add-on treatment to standard cognitive remediation approaches and such interventions have been aimed at driving adaptive plastic changes throughout brain areas relevant to cognition. These neuroplasticity-based training approaches have preliminarily proven some efficacy. A very recent machine learning contribution [122] revealed that the efficacy of a 10-hour neuroplasticity-based computerized cognitive training administered to Recent Onset Psychosis individuals could be predicted by resting-state functional connectivity features. More specifically, individuals who exhibited improvements in the attention domain after the training had a more intact sensory processing resting-state network at baseline. In these individuals, the cognitive gain was present despite the psychosis status. This finding encourages the investigation of non-pharmacological treatment response neuro-markers, as well as of their relationship with cognitive improvements potentially associated with neuroplasticity processes.

While taking into account subject- and practice-specific factors as potential predictors of outcomes, the effort to individualize cognitive rehabilitation should also take advantage of approach specificities. The list of rehabilitative strategies reported by studies we examined includes perception-based, music-and-art-based, psychosocial, yoga, and physical exercise-based approaches. Overall, the data we examined indicate that all these strategies are effective in enhancing cognitive performance in patients with SCZ, with CR and perception-based approaches producing rapid positive effects on cognition and most of the other approaches yielding long-term benefits on daily-life skills. In this light, CR/perception-based strategies could be combined with the other approaches in order to guarantee both short- and long-term benefits on the patient’s global level of functioning. Moreover, even though the pro-cognitive effects of most of these strategies may involve the same core mechanism of neuroplasticity, diversity among them should be treasured in tailoring rehabilitative intervention on patient’s specific strengths and weaknesses.

## CONCLUSION

Taken together, the studies we examined suggest that targeting cognitive impairments of SCZ in an individualized perspective is feasible only when an accurate identification of both individual-specific and approach-specific factors is present. This accurate identification allows clinicians to match patient’s specific needs and difficulties with the specific strength points of each and every available approach. Future studies aimed at systematically developing and distributing demographically stratified administration recommendations taking into account specific types and levels of cognitive deficits are strongly warranted.

## Figures and Tables

**Fig. (1):**
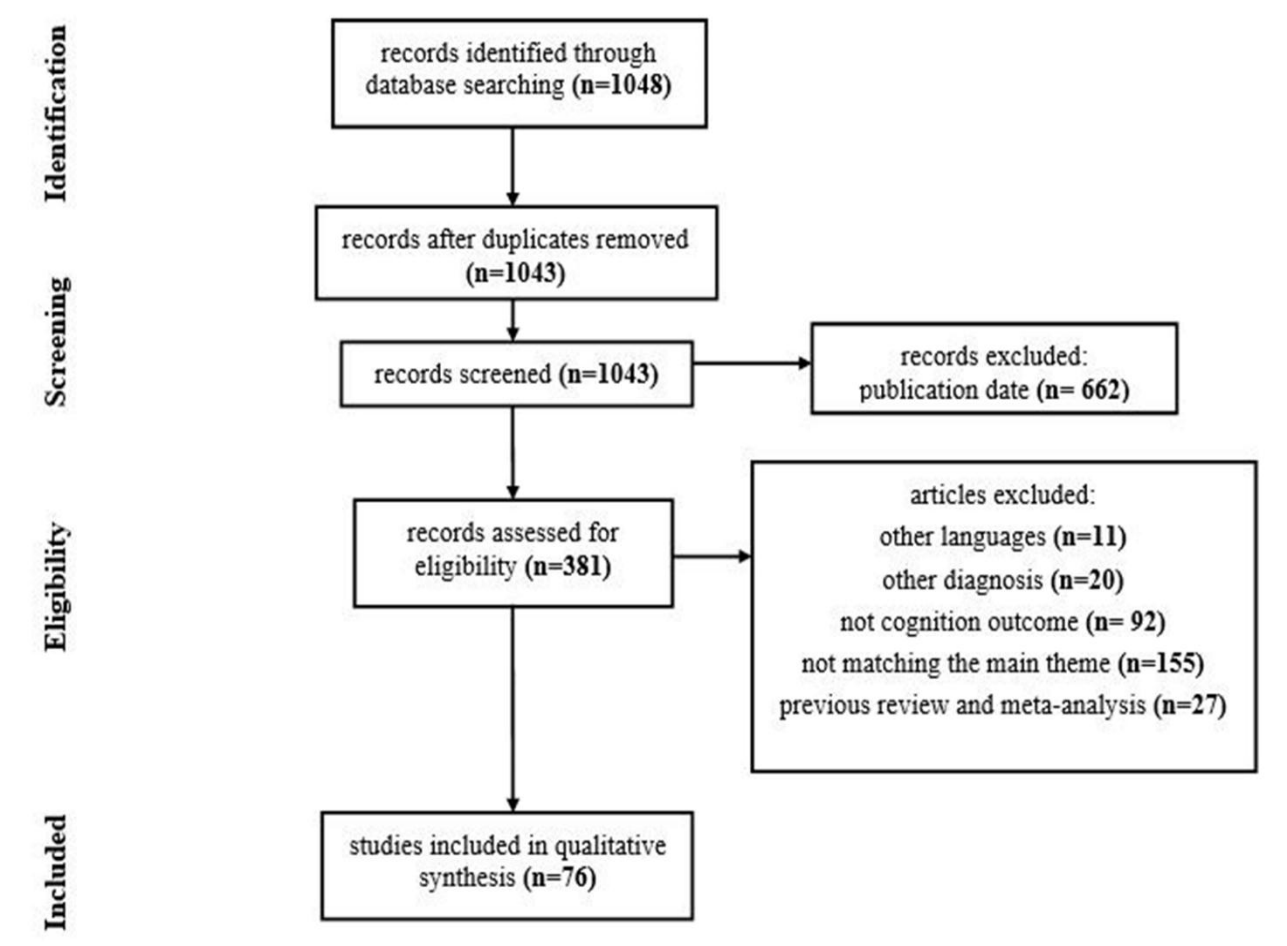
PRISMA flowchart of the review process.

**Table 1:** Summary of studies that met all inclusion criteria. For each study, the table reports details about study design, sample size, rehabilitative approaches adopted, and measures used to assess cognitive, clinical, and global functioning outcomes.

**Reference**	**Study Design**	**Diagnosis**	**Sample Size**	**Type of Intervention**	**Outcomes**
Kärgel *et al.* (2016)	Controlled clinical trial	SCZ/ SAD	46	Visual-Spatial Attention Training; Auditory Discrimination Training.	MMN (EEG)
Penadés *et al.* (2016)	Controlled clinical trial	SCZ	50	Cognitive Remediation Therapy vs control psychological treatment (Social Skills Training).	Digit Span, Letter-Number Sequencing and Arithmetic (WAIS-III), Digit Symbol-Coding (WAIS-III), TMT-A,TMT-B, RAVLT, Logical Memory I and II (WMS-III), Visual Reproduction I and II and Faces I and II (WMS-III), WCST, TOL, Cortical thickness.
Verma *et al.* (2018)	Controlled clinical trial	SCZ	21	Integrated yoga module for SCZ.	UKU, TMT-A, TMT-B, BPRS, SAPS, SANS, systolic blood pressure, diastolic blood pressure, BMI, waist circumferences, hip circumferences.
Woodward *et al.* (2018)	Controlled clinical trial	SCZ and SAD	17	Moderate-intensity exercise.	PANSS, SOFAS, ESRS, cardiovascular fitness, WTAR, KBIT, Hippocampal volume.
Zhang *et al.* (2015)	Multicenter, parallel-group, randomized controlled trial	SCZ (DSM-IV)	234	Comprehensive family therapy group vs the usual daily care group.	RBANS, PANSS.
Jørgensen *et al.* (2015)	Open randomized parallel group trial	SCZ or SAD	93	Guided Self-Determination group + Therapy As Usual vs Therapy As Usual alone.	BCIS, RAS, RSES, GAF, PANSS.
Kern *et al.* (2017)	Comparison between 2 controlled clinical trials	Veterans Affair study: SCZ or SAD	71	Errorless learning plus ongoing IPS (Individual Placement and Support model) supported employment vs ongoing IPS supported employment alone.	WBI, MCCB, BPRS.
Khazaal *et al.* (2015)	Multicentre randomized controlled trial	Psychotic disorder (DSM-IV)	172	Michael’s game + Therapy As Usual vs Therapy As Usual.	PDI-21, BPRS, BCIS, GAF, SOFAS, MADRS, Belief flexibility
Kimhy *et al.* (2015)	Single-Blind, Randomized Clinical Trial	SCZ or related disorders	33	Aerobic Exercise program utilizing active-play video games (Xbox 360 Kinect) vs traditional Aerobic Exercise equipment + Therapy As Usual.	Serum BDNF levels, SAPS, SANS, BDI, BAI, WTAR
Kurylo *et al.* (2018)	Controlled clinical trial	SCZ or SAD	28	Computer training vs Instrumental Enrichment therapy vs Computer-Assisted Cognitive Rehabilitation Therapy.	VOT, Perceptual organisation ability, Contour Integration Test, Picture Completion, Visual Discrimination, Brief Visual-Spatial Memory, Spatial Span (WAIS), Letter Number Sequencing, Dots Test, TMT-A, Matrix Reasoning (WAIS), Category Fluency, RFFT, Wide Range Achievement Test: Reading.
Lindenmayer *et al.* (2018)	Controlled clinical trial	SCZ or SAD	131	Computer-Assisted Cognitive Rehabilitation Therapy + Mind Reading Interactive Guide to Emotions; Brain Fitness alone; Brain Fitness + Mind Reading Interactive Guide to Emotions.	MCCB, FEIT, FEDT, ER-40, PSP, PANSS.
Peña *et al.* (2015)	Randomized clinical trial	SCZ	50	Film analysis.	PSP, TMT, BACS, HVLT, BVLT, WAIS-III, FAS, MSCEIT, Mind reading, Faux pas recognition.
Rakitzi *et al.* (2016)	Randomized controlled trial, the first RCT study for the efficacy of IPT in Greece.	SCZ	48	Integrated Psychological Therapy (IPT) vs Therapy As Usual.	CPT, LNS, Greek VMT, SPS, PANSS, WHOQOL, GAF.
Sohn *et al.* (2016)	Clinical trial	SCZ (DSM-IV)	9	Virtual Reality scenarios.	CGI-S, CGI-I, HAM-D, ZDRS, BAI, WCST, RCFT.
Su *et al.* (2016)	Single-blind, parallel assignment, randomized controlled design.	SCZ or SAD (DSM-IV)	57	Aerobic exercise vs stretching and toning control group.	PANSS, IQ, WAIS-III, MCCB, TMT-A, CPT-IP, WMS-III, WAIS-III, BVMT-R, NAB-mazes subtest, cardiorespiratory fitness.
Yang *et al.* (2018)	Clinical trial	SCZ	41	Music intervention + Therapy As Usual vs Therapy As Usual only.	BDT, BVRT, Spatial Maze Test, Resting-state fMRI.
Yildiz *et al.* (2019)	Clinical trial	SCZ or SAD	20	Psychosocial Skills Training or Metacognitive Training.	PANSS, CGI-S, GAF, QLS, CAI.
Kurtz *et al.* (2017)	Randomized clinical trial	SCZ or SAD	61	Story Method condition; Imagery condition vsControl condition.	HVLT-R, RBMT.
Lexen *et al.* (2015)	Cross-sectional study	SCZ	39	Vocational rehabilitation.	MCCB, TMT-A, WAIS-III, Visual Reproduction 1 and 2 subscales (WMS-III), TOL, BPRS.
Morimoto *et al.* (2018)	Controlled, randomized clinical trial	SCZ (DSM-IV)	31	Cognitive Remediation Therapy vs Therapy As Usual.	T1-weighted MRI, PANSS, BACS-J, LASMI
Mullen *et al.* (2017)	Clinical trial	BD I/II, Dystimic Disorder, MDD, AD, NOS SAD, SCZ	72	Compensatory Cognitive Training.	MCCB, CSEI
Perez *et al.* (2017)	Cross-sectional study	SCZ (DSM-IV)	28	Auditory Perceptual Training Exercises.	SANS, SAPS, MMN (EEG).
Ramsay *et al.* (2017)	Triple-blind trial	SCZ or SAD (DSM-IV)	85	Cognitive Remediation Therapy vs Computer Skills Training.	MCCB, N-back, UPSA, SSPA, BPRS, fMRI.
Spangaro *et al.* (2018)	Retrospective study	SCZ (DSM-IV)	88	Cognitive Remediation Therapy + Standard Rehabilitation Therapy.	PANSS, WAIS-R, WCST
Subramaniam *et al.* (2017)	Randomized clinical trial	SCZ, SAD, or NOS Psychoses	76	Targeted Cognitive Training.	PANSS, MCCB
Kukla *et al.* (2018)	Single-blind, three-armed randomized controlled trial	SCZ or SAD	75	Vocational support vs work-focused Cognitive Behavioral Therapy vs work-focused Cognitive Behavioral Therapy enhanced with Cognitive Rehabilitation.	WBI, MCCB, PANSS
Kurtz *et al.* (2015)	Clinical trial	SCZ or SAD	64	Social Skills-Training (SST) Cognitive Remediation vs Computer Skills Training.	WAIS-III or IV, PCPT, CVLT-II, PCET, SSPA
Lindenmayer *et al.* (2017)	Clinical trial	SCZ or SAD	63	Cognitive Remediation.	MCCB, PSP, SOFAS, PANSS
Lystad *et al.* (2016)	Clinical trial	SCZ spectrum disorder	122	Cognitive Behavioral Therapy + Vocational rehabilitation vs Cognitive Remediation + Vocational rehabilitation.	M.I.N.I. plus, PANSS, WASI, MCCB
Matsuda *et al.* (2016)	Clinical trial	SCZ	62	Cognitive Remediation.	BACS-J, PANSS, LASMI, JART
Pena *et al.* (2018)	Parallel-group randomized trial	SCZ (DSM-IV]	101	REHACOP (Neurocognitive remediation + Social Cognitive Intervention + functional skills training].	UPSA, GAF, WAIS-III, MSCEIT
Schutt *et al.* (2017)	Clinical trial	SCZ or SAD	6	Cognitive Enhancement Therapy.	MCCB, UPSA
Thomas M *et al.* (2019)	Clinical trial	SCZ or another psychotic disorder	53	Cognitive Remediation Therapy.	RBANS Update, ABAS-3, GAS
Thomas KR *et al.* (2016)	Clinical trial	SCZ and SADs, bipolar and major depressive disorder (DSM-IV)	59	Compensatory Cognitive Training.	BACS, CPT-IP, WMS-III, BVMT-R, HVLT-R, Mazes (NAB), WCST-64, SSPA, ILSS, QOLI, HAM-D, PANSS, GDS
Thomas ML *et al.* (2018)	Clinical trial	SCZ or SAD (DSM-IV)	46	Targeted cognitive training + Therapy As Usual vs Therapy As Usual alone.	MATRICS, MCCB, SANS, SAPS
Tsang *et al.* (2016)	Clinical trial	SCZ or SAD	90	Integrated Supported Employment + Cognitive Remediation Therapy vs Integrated Supported Employment alone.	BPRS, GAF, MCCB
Twamley *et al.* (2017)	Clinical trial	SCZ, SAD, BD, MDD	153	Compensatory Cognitive Training vs Enhanced Supported Employment.	Job attainment, hours worked, wages earned, UPSA, HAM-D, ILSS, QoLI, TMT, BACS, HVLT-R, BVMT-R, CPT-IP, NAB, FAS, MIST
Bryce *et al.* (2018)	Assessor-blind RCT	SCZ or SAD	56	Cognitive Remediation VS Computer Game	MATRICS, MCCB, PANSS, RSES, QLS, ILSS-SR.
Bora *et al.* (2016)	Cross-sectional study	SCZ or BD	124	No intervention.	SCWT, WCST, ToM: RMET, Hinting task
Davidson *et al.* (2016)	RCT	SCZ	75	Cognitive Remediation + Compensatory Cognitive Training vs Therapy As Usual.	CVLT-II
Farreny *et al.* (2016)	RCT	SCZ or SAD	62	Cognitive Remediation vs stimulating activities focused on leisure and socialization.	BADS
Gabbatore *et al.* (2017)	Single case report with experimental design	SCZ	Single case.	Cognitive Pragmatic Treatment.	CPT
Ikebuchi *et al.* (2017)	Multisite, individual-level RCT	SCZ, BD or MDD	94	Cognitive remediation + Supported Employment vs Traditional Vocational Services.	Competitive employment rates, total days employed, total earnings during the follow-up period, BACS, PANSS, HAM-D, GAF, LASMI
Jashan *et al.* (2019)	RCT	SCZ or SAD	99	Brain Fitness.	MCCB, UPSA, MMN (EEG)
Kumar *et al.* (2019)	Quasi-experimental intervention design	SCZ	34	Cognitive Remediation Therapy.	Working memory, ToL, Complex figure test for visuo-spatial memory, WCST, SANS, SAPS
Ahmed *et al.* (2015)	assessor-blind RCT	SCZ or SAD	78	Cognitive Remediation Therapy.	WASI-II, MATRICS, PANSS, OAS, UPSA, MARS
Au *et al.* (2015)	assessor-blind RCT	SCZ or SAD	90	Integrated Supported Employment vs Cognitive Remediation Therapy.	BPRS, GAF, RSES, WCST, MCCB, HKLLT-2
Bechi *et al.* (2015)	RCT	SCZ	75	Computer-Assisted Cognitive Rehabilitation Therapy.	Total Sequencing score, Total Questionnaire score.
Buonocore *et al.* (2017)	Monocentric retrospective study	SCZ	60	Computer-Assisted Cognitive Remediation Therapy vs Standard Rehabilitation Therapy.	PANSS, BACS, QLS
Buonocore *et al.* (2017)	Randomized single blind trial, followed by an open label trial	SCZ	129	Standard Rehabilitation Treatment + Computer-Assisted Cognitive Remediation Therapy.	PANS, BACS, WAIS–R, QLS
Buonocore *et al.* (2018)	Monocentric retrospective study	SCZ	60	Standard Rehabilitation Therapy + Socio-Cognitive Rehabilitation.	PANSS, PAS, WAIS, BACS, Total Questionnaire score.
Corbera *et al.* (2015)	Retrospective study → secondary data analysis from two, blinded RCTs	SCZ or SAD	112	Cognitive Remediation Therapy,	Digit-Span, Letter-Number-Sequencing subtests (WAIS-III & IV), PANSS, UPSA-B
Deste *et al.* (2019)	Retrospective study	SCZ	56	Computer-Assisted Cognitive Remediation Therapy vs Integrated Psychological Therapy.	CGI-S, PANSS, TMT-A, TMT-B, WCST, SOPT, CVLT, GAF, HoNOS
Deste *et al.* (2015)	Retrospective study	SCZ	84	Integrated Psychological Therapy or Computer-Assisted Cognitive Remediation Therapy vs Treatment As Usual.	PANSS, HoNOS.
Donohoe *et al.* (2017)	Single blind RCT	SCZ	90	Cognitive Remediation Therapy.	SAPS, SANS, WMS-III, LNS, CANTAB, WASI, SOC (CANTAB), WCST, Stroop test, TMT, ToM: RMET, SOFAS, UPSA, RSES, ILS, WHOQoL
Fiszdon *et al.* (2016)	RCT	SCZ or SAD	75	Cognitive Remediation Therapy.	WAIS, TMT-A, TMT-B, CVLT-II, WMS-R, RCFT, WAIS-III, FAS, CPT, PANSS, UPSA, SSPA, MMAA, ILSS, QLS
Garrido *et al.* (2017)	Retrospective study on a single-blind RCT	SCZ	33	Computer-Assisted Cognitive Remediation Therapy.	PANSS, SDMT, FAS, WAIS-III, CVLT, WCST-CV3, Stroop Test, Matrix Reasoning subtest (WAIS-III), QLS, RSES, PES, APU
Iwata *et al.* (2017)	Multicenter RCT	SCZ	60	Cognitive Remediation Therapy vs Therapy As Usual.	BACS, LASMI, PANSS
John *et al.* (2017)	Retrospective naturalistic study	SCZ	89	Psychosocial Rehabilitation Program.	BACS
Katsumi *et al.* (2017)	RCT	SCZ or SAD	44	Cognitive Remediation using NEAR (neuropsychological educational approach] vs Therapy as Usual.	JART, PANSS, BACS, GAF
Bryce *et al.* (2018)	Single-blind RCT	SCZ or SAD	49	Computer-Assisted Cognitive Rehabilitation Therapy	MCCB, PANSS, IMI-SR
Cerino *et al.* (2020]	Not randomized not controlled trial.	SCZ or SAD	10	Integrated Psychological Therapy + Computer-Assisted Cognitive Rehabilitation Therapy.	RBANS, MCST
Bell *et al.* (2018)	RCT	SCZ or SAD	77	Vocational Rehabilitation vs Vocational Rehabilitation + Computer Games.	PANSS, QLS, Job attainment, Volunteer work, WTAR, MATRICS
Fardig *et al.* (2016)	Retrospective study	SCZ or SAD	53	Illness Management and Recovery Program.	PECC
Fisher *et al.* (2017)	Ongoing, double-blind RCT	SCZ, SAD, NOS Psychoses	111	Targeted auditory and visual system training + intensive social cognitive training vs Targeted auditory and visual system training alone.	MCCB, MSCEIT, PROID, Faux Pas Recognition Test, TEPS, PANSS, UPSA-B, QLS, SFS
Bathia *et al.* (2017)	Single-blind RCT	SCZ	340	Supervised yoga training + Therapy As Usual or Supervised physical exercise training + Therapy As Usual vs Therapy As Usual alone.	Penn CNB, SAPS, SANS.
Buonocore *et al.* (2015)	Assessor-blind RCT	SCZ	57	Metacognitive group training vs Computer-Assisted Cognitive Remediation Therapy.	PANSS, BACS, BADE
Aloi *et al.* (2018)	Assessor-blind RCT	SCZ	41	Cognitive-behavioral therapy.	PANSS, GAF, WHOQOL.
Buonocore *et al.* (2017)	Retrospective study	SCZ (DSM-IV-TR]	104	Computer-Assisted Cognitive Remediation Therapy vs Standard Rehabilitation Therapy.	PANSS, WAIS-R, BACS, Picture Sequencing Task, QLS
Byrne at al. (2015)	Not randomized controlled clinical trial	SCZ	40	Cognitive Remediation Computerized Drill Training vs Therapy As Usual.	PANSS, CGI, PSP, LNS, HKLLT-B.
Contreras *et al.* (2017)	Assessor-blind RCT	SCZ	20	Cognitive Remediation Therapy + Visual Processing Training.	MCCB, SERS-SF, EUROHIS-QoL, FS, WTAR, PANSS, SANS, SAPS
Firth *et al.* (2016)	Not-randomized feasibility trial	First Episode Psychosis	31	Aerobic Exercise.	PANSS, BDI-II, SIAS, SOFAS, WHODAS 2.0, WHOQoL-B, 6-Minute Walk test, IPAQ, TMT-A, TMT-B, Digit-Symbol Coding, SOC, Spatial span, Erikson Flanker Task, Finger tapping, Motor screening, ToM: RMET.
Horan *et al.* (2017)	Assessor-blind RCT	SCZ, SAD, NOS Psychoses	139	Psychiatric Rehabilitation.	MCCB, BPRS, UPSA, MASC, RFS
Fiszdon *et al.* (2016)	Open, uncontrolled, proof-of concept trial	SCZ	58	Cognitive Remediation Therapy.	MCCB, PANSS, QLS, IMI–SR, USSST, IPSAQ, AIHQ, TASIT, DACOBS, BLERT
Gomar *et al.* (2015)	Assessor-blind RCT	SCZ or SAD	130	Computer-Assisted Cognitive Training or Computerized Active Control condition vs Treatment As Usual.	BADS, RBMT, UPSA, WMS, Stroop Test, TMT, FAS, DEX, MCL
Dubreucq *et al.* (2020)	Controlled, quasi-experimental, multi-centric, prospective, interventional and exploratory trial	SCZ	87	Remed Rugby (a behavioral training intervention targeting cognitive deficits).	PSP, MASC, AIHQ, QCAE, ERF-CS, PANSS, ISMI, SERS-SF, WAIS, TMT-A, TMT-B, BEM-144 -SS, NART

## LIST OF ABBREVIATIONS

SCZ: = Schizophrenia;
SAD: = Schizoaffective Disorder;
BD: = Bipolar Disorder;
NOS: = Not Otherwise Specified;
MDD: = Major Depressive Disorder;
AD: = Anxiety Disorder;
ABAS-3: = Adaptive Behavior Assessment System Third Edition;
AIHQ: = Ambiguous Intentions Hostility Questionnaire;
APU: = Acute Psychiatric Unit;
BACS: = Brief Assessment of Cognition in SCZ;
BACS - J: = Brief Assessment of Cognition in SCZ - Japanese version;
BADE: = Bias Against Disconfirmatory Evidence;
BADS: = Behavioral Assessment of the Dysexecutive Syndrome;
BAI: = Beck Anxiety Inventory;
BCIS: = Beck Cognitive Insight Scale;
BDI: = Beck Depression Inventory;
BDT: = Behavior Driven Testing;
BEM 144 - SS: = Batterie d'Efficince Mnesique 144 - Story Subtest;
BLERT: = Bell-Lysaker Emotion Recognition Task;
BMI: = Body Mass Index;
BPRS: = Brief Psychiatric Rating Scale;
BVLMT: = Brief Visual Learning and Memory Test;
BVMT-R: = Brief Visuospatial Memory Test-Revised;
BVRT: = Benton Visual Retention Test;
CAI: = Cognitive Assessment Interview;
CANTAB: = Cambridge Neuropsychological Test Automated Battery;
CDS: = Conduct Disorder Scale;
CGI: = Clinical Global Impressions;
CGI-I: = Clinical Global Impressions – Improvement;
CGI-S: = Clinical Global Impressions – Severity;
CPT: = Continuous Performance Test;
CPT-IP: = Continuous Performance Test - Identical Pairs;
CSEI: = College Self-Efficacy Inventory;
CVLT-II: = California Verbal Learning Test -II;
DACOBS: = Davos Assessment of Cognitive Biases Scale;
DEX: = Dysexecutive Questionnaire;
ER-40: = Penn Emotion Recognition Task;
ERF-CS: = Social Cognition-Functional Outcomes Scale;
ESRS: = Extrapyramidal Symptoms Rating Scale;
QOL: = European Health Interview Survey - Quality of Life;
FAS: = Verbal Fluency Test;
FEDT: = Facial Emotion Discrimination Test;
FEIT: = Facial Emotion Identification Test;
fMRI: = functional Magnetic Resonance Imaging;
FS: = Friendship Scale;
GAF: = Global Assessment of Functioning;
GAS: = Goal Attainment Scaling;
GDS: = Geriatric Depression Scale;
HAM-D: = Hamilton Rating Scale for Depression;
HKLLT-2: = Hong Kong List Learning Test – 2;
HKLLT-B: = Hong Kong List Learning Test - form B;
HoNOS: = Health of the Nation Outcome Scale;
HVLMT: = Hopkins Verbal Learning and Memory Test;
HVLT-R: = Hopkins Verbal Learning Test – Revised;
ILS: = Independent Living Scales;
ILSS: = Independent Living Skills Survey;
ILSS: = Independent Living Skills Survey;
ILSS-SR: = Independent Living Skills Survey - Self Report;
IMI-SR: = Intrinsic Motivation Inventory for SCZ Research;
IPAQ: = International Physical Activity Questionnaire;
IPSAQ: = Internal Personal and Situational Attributions Questionnaire;
IQ: = Intelligence Quotient;
ISMI: = Internalized Stigma of Mental Illness;
JART: = National Adult Reading Test Japanese version;
KBIT: = Kauffmann Brief Intelligence Test;
LASMI: = Life Assessment Scale for the Mentally;
LNS: = Letter Number Span;
M.I.N.I - Plus: = Mini International Neuropsychiatric Interview – Plus;
MADS: = Mania Diagnostic and Severity Scale;
MARS: = Maryland Assessment of Recovery in Severe Mental Illness;
MASC: = Maryland Assessment of Social Competence;
MCCB: = MATRICS Consensus Cognitive Battery;
MCL: = Memory Checklist;
MCST: = Modified Card Sorting Test;
MIST: = Memory for Intentions Screening Test;
MMAA: = Medication Management Ability Assessment;
MMN: = Mismatch Negativity;
MSCEIT: = Mayer-Salovey-Caruso Emotional Intelligence Test;
NAB: = Neuropsychological Assessment Battery;
NART: = National Adult Reading Test;
OAS: = Overt Aggression Scale;
PANSS: = Positive And Negative Syndrome Scale;
PAS: = Premorbid Adjustment Scale;
PCET: = Penn Conditional Exclusion Test;
PCPT: = Penn Continuous Performance Test;
PDI-21: = Peters *et al.* Delusions Inventory – 21;
PECC: = Psychosis Evaluation tool for Common use by Caregivers;
Penn CNB: = University of Pennsylvania Computerized Neurocognitive Battery;
PES: = Psychiatric Emergency Services;
PROID: = Prosody Identification Test;
PSP: = Personal and Social Performance Scale;
QCAE: = Questionnaire of Cognitive and Affective Empathy;
QLS: = Quality of Life Scale; QoLI= Quality of Life Inventory;
RAS: = Recovery Assessment Scale;
RAVLT: = Rey Auditory Verbal Learning Test;
RBANS: = Repeatable Battery for the Assessment of Neuropsychological Status;
RBMT: = Rivermead Behavioural Memory Test;
RCFT: = Rey-Osterrieth Complex Figure Test;
RFFT: = Ruff Figural Fluency Test;
RFS: = Role Functioning Scale;
RSES: = Rosenberg Self-Esteem Scale;
RSES: = Revised Self-Efficacy Scale;
SANS: = Scale for the Assessment of Negative Symptoms;
SAPS: = Scale for the Assessment of Positive Symptoms;
SCWT: = Stroop Color World Test;
SDMT: = Symbol Digit Modalities Test;
SERS-SF: = Self-Esteem Rating Scale - short form;
SFS: = Social Functioning Scale;
SIAS: = Social Interaction Anxiety Scale;
SOC: = Stockings of Cambridge task;
SOFAS: = Social Occupational and Functional Assessment Scale;
SOPT: = Self-Ordered Pointing Task;
SPS: = Social Perception Scale;
SSPA: = Social Support for Physical Activity;
TASIT: = The Awareness of Social Inference Test;
TEPS: = Temporal Experience of Pleasure Scale;
TMT-A: = Trail Making Test – A;
TMT-B: = Trail Making Test – B;
TOL: = Tower of London;
ToM: = RMET= Theory of Mind: Reading the Mind in the Eyes Test;
UKU-SERS: = UKU Side Effect Rating Scale;
UPSA: = UCSD Performance-Based Skills Assessment;
UPSA-B: = UCSD Performance-Based Skills Assessment - Brief Version;
USSST: = Understanding Social Situations Skills Test;
VMT: = Verbal Memory Test;
VOT: = Hooper Visual Organization Test;
WAIS: = Wechsler Adult Intelligent Scale;
WAIS-III: = Wechsler Adult Intelligent Scale – III;
WAIS-IV: = Wechsler Adult Intelligence Scale IV;
WAIS-R: = Wechsler Adult Intelligence Scale Revised;
WASI: = Wechsler Abbreviated Scale of Intelligence;
WASI-II: = Wechsler Abbreviated Scale of Intelligence II;
WBI: = Well-Being Inventory;
WBI: = Well-Being Inventory;
WCST: = Wisconsin Card Sorting Test;
WCST-64: = Wisconsin Card Sorting Test - 64 Cards Version;
WCST-CV3: = Wisconsin Card Sorting Test - Computer Version 3;
WHO QoL: = World Health Organization Quality of Life;
WHO QoL-B: = World Health Organization Quality of Life - Brief Version;
WMS-III: = Wechsler Memory Scale;
WMS-R: = Wechsler Memory Scale Revised;
WTAR: = Wechsler Test of Adult Reading;
ZDRS: = Zung Depression Rating Scale
